# State of the art in tracheal surgery: a brief literature review

**DOI:** 10.1186/s40248-018-0147-2

**Published:** 2018-09-12

**Authors:** Alessandra Siciliani, Erino Angelo Rendina, Mohsen Ibrahim

**Affiliations:** Department of Thoracic Surgery, Sant’Andrea Hospital, Sapienza University of Rome, Rome, Italy

**Keywords:** Tracheal surgery, Laryngotracheal resection, Subglottic stenosis, Anastomotic complications

## Abstract

**Background:**

Tracheal surgery requires a highly specialized team of anesthesiologists, thoracic surgeons, and operative support staff. It remain a formidable challenge for surgeons due to the criticality connected to anatomical considerations, intraoperative airway management, technical complexity of reconstruction, and the potential postoperative morbidity and mortality.

**Main body:**

This article focuses on the main technical aspects and literature data regarding laryngotracheal and tracheal resection and reconstruction. Particular attention will be paied to anastomotic and non-anastomotic complications.

**Short conclusion:**

Results from literature confirm that, when feasible, laryngotracheal and tracheal resection and reconstruction is the treatment of choice in cases of benign stricture and malign neoplasm. Careful patient selection, operative planning, and execution are required for optimal results.

## Background

The first tracheal surgical procedures described date back to the second and third century with the reports of Aretaeus and Galen on tracheostomy. Despite this ancient acknowledgment, modern tracheal surgery developed much later. In 1950, Barclay described the first tracheal resection [1]. It was only in 1990 that Grillo demonstrated the feasibility of surgical treatment of tracheal stenosis for the first time and later, surgery for any type of tracheal disease requiring resection, including tumors, by resection of a portion of the trachea and its reconstruction by primary reanastomosis [2, 3].

The hyoid bone, larynx, cricoid and trachea compose the upper airway. The trachea is a cartilaginous and membranous airway extending from the lower larynx to the carina and is approximately 11 cm in length and 2–2.5 cm in diameter. The subglottic space extends from the inferior margin of the vocal cords to the lower border of the cricoid cartilage and represents the narrowest part of the airway. The laryngeal nerves enter the cricoid in its posterior portion and the resection of the entire cricoid is impossibile without damaging both recurrent nerves destroying their protective function. These anatomical characteristics make the trachea a rigid and unextendable structure, for that, tracheal and laryngotracheal surgery remain at date a challenge for the thoracic surgeon.

Laryngotracheal (LTRR) and tracheal resection and reconstruction (TRR) will be discussed in this article.

## Main text

Interventional pulmonology treatments, such as mechanical dilatation, laser ablation and stenting have a limited and transient role in the treatment of tracheal lesions due to frequent recurrences. As described by Brichet in 1999, only 17.6% of complex tracheal stenoses treated with laser ablation and stenting achieved satisfactory results [4]. Galluccio et al., similarly analyzed the results of their large series of subglottic stenosis treatment and confirmed that endoscopic treatment of complex subglottic stenoses with lesions > 1 cm and involvement of the tracheal wall is contraindicated and, when feasible, surgery should remain the treatment of choice [5]. For many years, temporary Montgomery T-tube placement and tracheostomy were considered the only possible alternatives to surgery. Those treatments are now discouraged due to the risk of bacterial colonization and extension of the diseased segment. Repeated procedures also are not recommended due to the risk of devascularization [6]. Some authors have reported a successful rate of 100% in cases of web-like stenosis and in few cases of complex stenosis, treated with endoscopic treatment as laser and stenting, and recommended these treatment in cases of high risk patients or excessive length stenosis not suitable for surgery [7, 8]. Bourinet et al. recently analyzed their experience with transcordal silicone stents in adult laryngotracheal stenosis reporting low morbidity and excellent clinical outcomes on long term follow up [9].

**Fig. 1:**
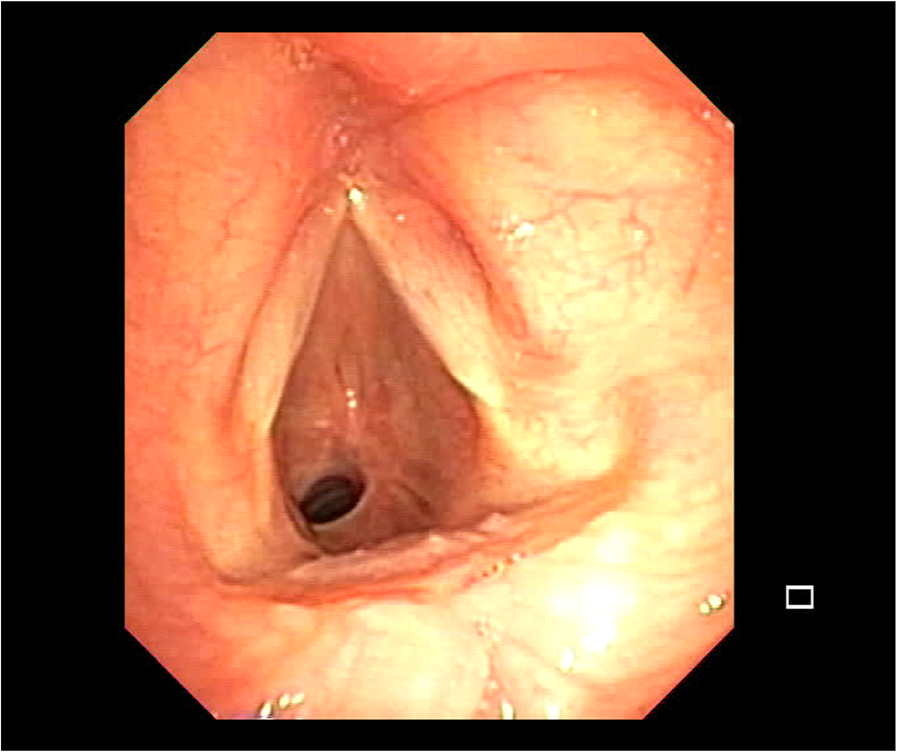
Endoscopic view of an idiopathic complex tracheal stenosis

The most common indications for LTRR and TRR are symptomatic concentric stenosis either idiopathic or related to prolonged intubation (Fig. 1). The innovation in respiratory intensive care units allow the management of patients with prolonged mechanical ventilation. The airway stenosis are caused by the pressure-induced ischemic injury of the tracheal wall due to endotracheal tubes with subsequent circumferential scarring and narrowing of the involved trachea. Tracheal stricture can occur also because of previous tracheostomy, tracheoesophageal fistula, post-traumatic lesions of malignancy. Clinical presentation is usually acute or chronic dyspnea and other symptoms may include cough, stridor and hemoptys. When symptoms occured, the trachea is usually narrowed up to 75% of its lumen [6]. Patients with subglottic stenosis and tracheostomy usually have bacterial colonization of the tracheostomy site and, according to Ciccone et al., systemic and local antibiotic treatment should be administered preoperatively [10].

Palliative endoscopic laser treatment is the only exception in those cases. Surgical resection currently is the curative treatment of choice.

**Fig. 2:**
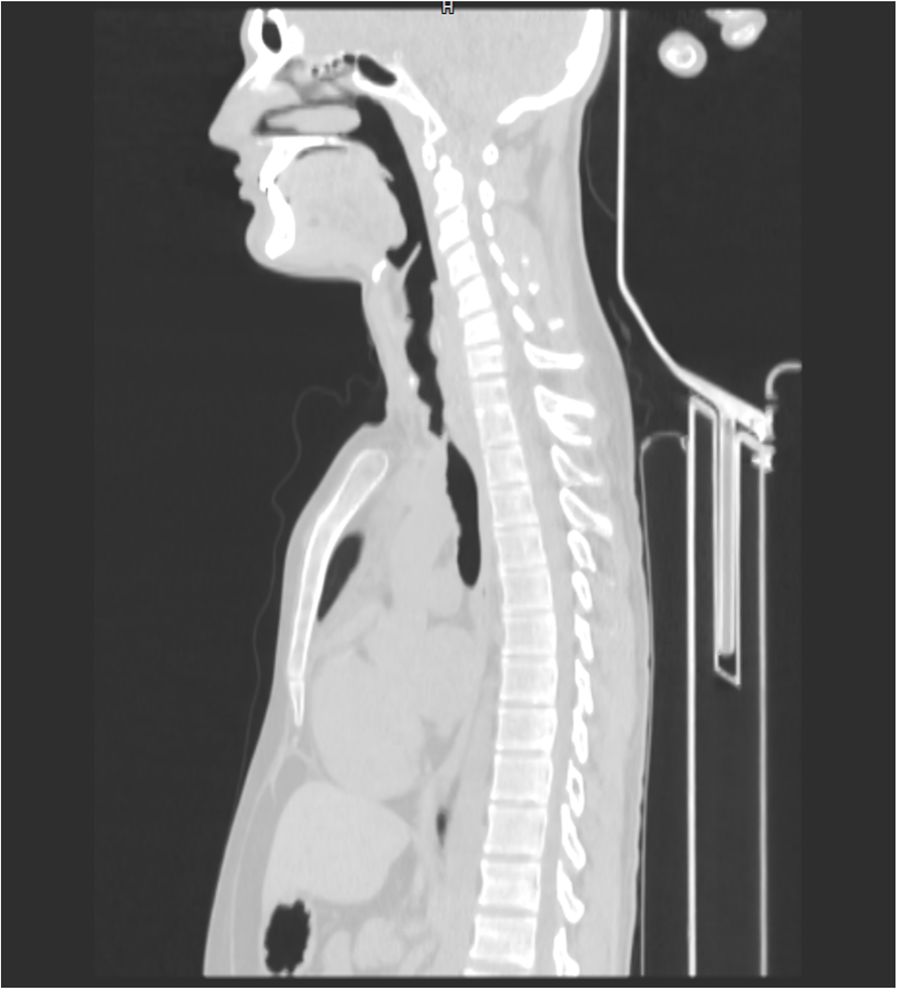
CT scan of tracheal stenosis

Preoperative evaluation includes clinical and radiological studies. Chest computed tomography (CT) with multiplane reconstruction is required (Fig. 2). Preoperative flexible bronchoscopy is mandatory for the study of the tracheal segments involved, mobility and integrity of the vocal cords, the severity and extent of longitudinal spread of disease, grade of inflammation, and presence of edema or malacia. In cases with a high degree of stenosis, sufficient subglottic space is required for a successful resection and reconstruction. Concomitant glottic pathology must be treated preoperatively. The technical challenge of airway resection is the extent of longitudinal spread. In 2004, Wright et al. recognized that surgical resections of benign lesions are optimally performed for segments 4 cm to 6 cm in length or at least 30% of the total tracheal length in children and 50% in adults [11]. Furthermore, Lancaster et al. considered resectable tumors less than 4 cm in length [12]. Wright et al. and Blasberg et al. emphasized that morbidity and mortality in tracheal surgery relate to anastomotic tension or devascularization. There is agreement in the literature that residual microscopic disease is permissible in order to avoid excessive tracheal resection [11, 13].

Relative contraindications to surgery include a history of local radiation treatment, previous tracheal surgery, mucosal inflammation beyond the area of resection, or ongoing high dose steroid therapy. Wright et al. noted that, when feasible, patients should be weaned from steroids 2 to 4 weeks before resection and the use of steroids after LTRR and TRR should be limited and allowed only in cases of severe glottic edema due to impairment of anastomotic healing [11]. In diabetic patients, medical therapy should be optimized preoperatively.

Close cooperation between the surgeon and anesthesiologist is fundamental in successful tracheal surgery. Total intravenous anesthesia is generally recommended. A single lumen armed tube is preferable since a double lumen endotracheal tube often presents difficulties from its inflexibility and size. Ventilation during the surgical procedures switches between different modes based on the surgical phase: cross-field ventilation, high jet ventilation (HFJV) [14], or oro-tracheal intubation. At the end of the reconstruction, the trans-field intubation tube is removed. The endotracheal tube is advanced into the trachea, and the cuff is reinflated. In 2010, Macchiarini et al. proposed airway surgery in awake, non-intubated patients under cervical epidural anesthesia [15]. Later authors, such as Loizzi et al. and Liu et al., confirmed that awake surgery in tracheal surgery is feasible [16, 17]. However, non–intubated anesthesia is discouraged by several authors due to a lack of evidence suggesting potential advantages in terms of perioperative management [18, 19]. Moreover, during the resection, it is vital to constantly reassess the ventilation, monitoring for hypoxia and hypercapnia. In traditional anesthesia, there is no consensus about the timing of extubation. Some authors prefer immediate extubation [11] while others advocate leaving the nasotracheal tube in place for 24 h in awake patients, with its tip distal to the anastomosis. Their rationale is protection of the suture, thus allowing a safe tracheobronchial toilette and extubation [18, 19].

## Surgical aspects-operative technique

The patient is placed in the supine position in the operating room and the neck is flexed posteriorly and hyperextended to help deliver the trachea out of the thoracic inlet and reduce anastomotic tension. Surgical approach depends on the localization and extent of the tracheal lesions. A cervical collar incision is usually performed on the upper third of the trachea; a cervical collar incision combined with partial or total sternotomy is preferred for lesions in the middle third of the trachea; and total sternotomy or fifth rigth thoracotomy is necessary for distal tracheal lesions [3, 12, 20].

### Resection and reconstruction techniques based on the site of lesions

In LTRR, when the disease involves the subglottic region near the vocal cords, there are many technical problems due to the necessity of extending the resection to the cricoid cartilage and the high risk of damaging both of the recurrent laryngeal nerves as experienced by Ogura and Powers in 1964 [21]. In 1974, Gerwat and Bryce overcame the problem using an oblique line to section the anterior cricoid arch and preserve the posterior cricoid plate. This technique has a limited role in treatment of posterior subglottic lesions [22]. In 1975, Pearson et al. [23] modified this approach with a transverse anterior resection of the subglottic airway a few millimeters below the vocal cords. They used primary thyro-tracheal end-to-end anastomosis performed near the vocal cords using interrupted sutures of 3–0/4–0 absorbable material placed in a concentric fashion 3 to 4 mm apart and 3 to 4 mm from the cut edge of the airway. Some authors preferred continuous 4–0 PDS sutures in the mucosal layer and interrupted 3–0 PDS sutures in the cartilaginous layer [24, 25]. They reported that traction sutures can be placed laterally above and below the anastomosis if necessary. The knots are then tied and laid outside the lumen. Size discrepancy can occur, but usually it is not necessary to tailor either end. This is the current technique of choice for most surgeons [18, 25–27, 41].

**Fig. 3:**
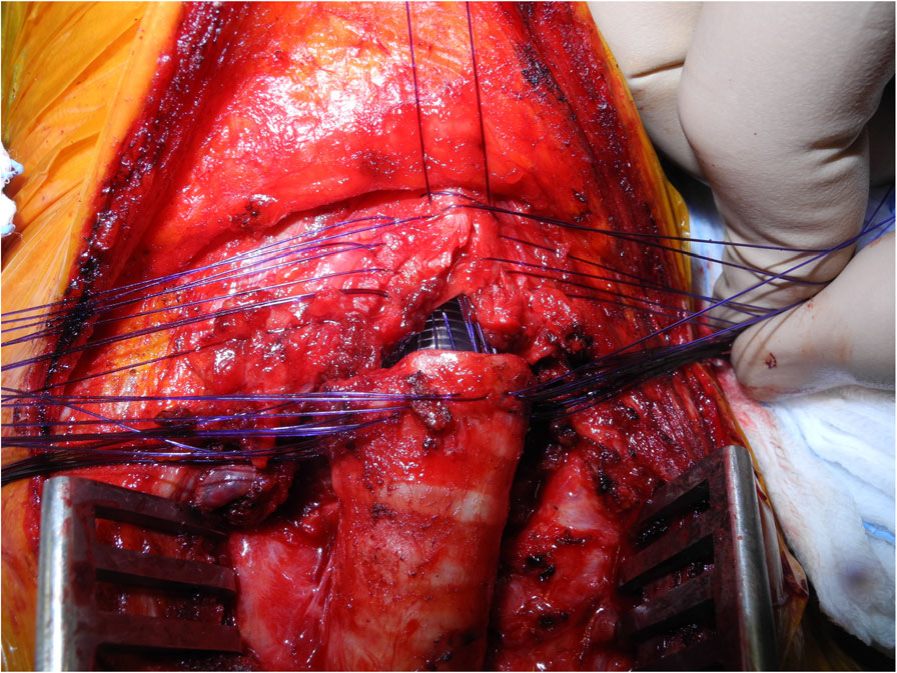
Introperatory view of laryngoplasty: the anastomosis is performed with an end-to-end interrupted suture in an outside-to-inside manner directly on the retracted ends of the thyroid cartilage

According to Maddaus et al. and Couraud et al., previous laryngoplasty is often required when the vocal cords are involved. Different techniques are available and usually include the resection of the anterior cricoid arch first and subsequent vertical division of the thyroid cartilage and the posterior cricoid plate [28, 29]. If necessary, an autologous tissue graft, bone or cartilage, can be inserted between the divided cartilaginous portions [30]. In 2016, Ciccone et al. presented a variation of the standard Pearson technique for subglottic LTRR [31]. The subglottic structure is resected with the anterior portion of the cricoid arch and the crico-thyroid membrane while a 1–2 cm laryngofissure is performed longitudinally to divide the thyroid cartilage in the midline. The margins are then retracted laterally to increase the airway space. The apex of the incision reaches the vocal cords anteriorly. The trachea is divided as usual and the anastomosis is performed with an end-to-end interrupted suture in an outside-to-inside manner directly on the retracted ends of the thyroid cartilage (Fig. 3). Reconstruction for stenosis or malignancy is accomplished in the same fashion eccept for the margin. Intraoperative frozen-section of the margins is required to achieve R0 resection.

Generally the dissection proceed circumferentially to the trachea, avoiding injury to the recurrent laryngeal nerves or entering the esophagus posteriorly. Attention must be payied to the blood supply too. The inferior thyroid artery supplies the upper trachea, while bronchial or intercostal arteries supply the lower trachea. Lateral dissection proximal and distal to the lines of resection should be limited for 1–2 cm to avoid devascularization af the airway.

**Fig. 4:**
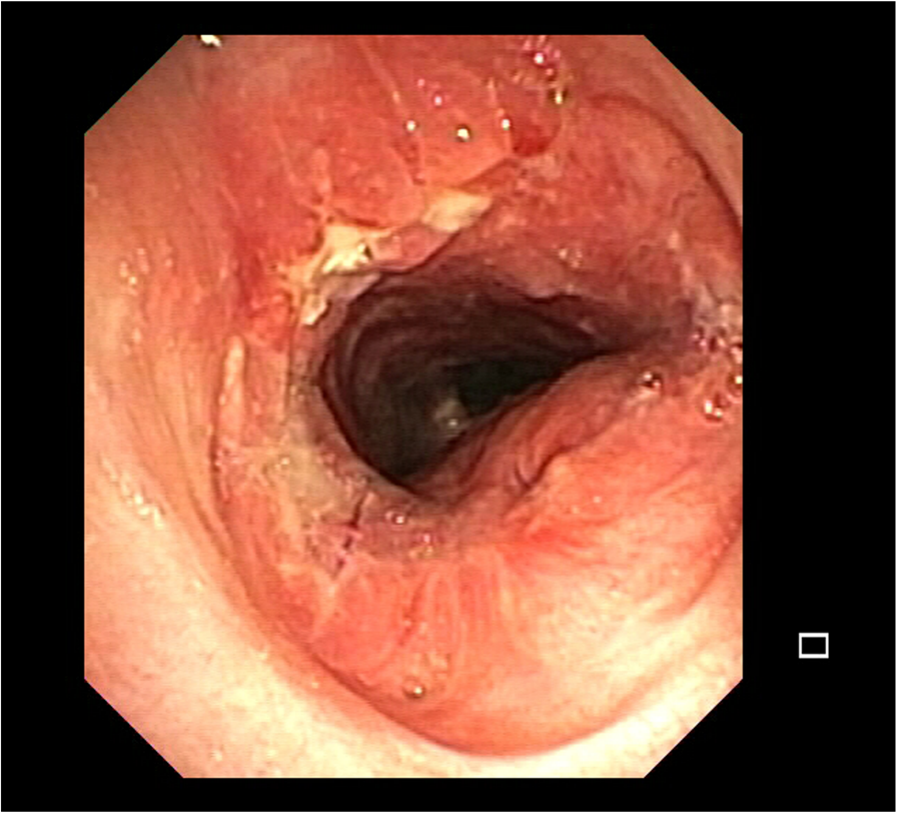
Endoscopic view of the anastomosis at the end of the operation

**Fig. 5:**
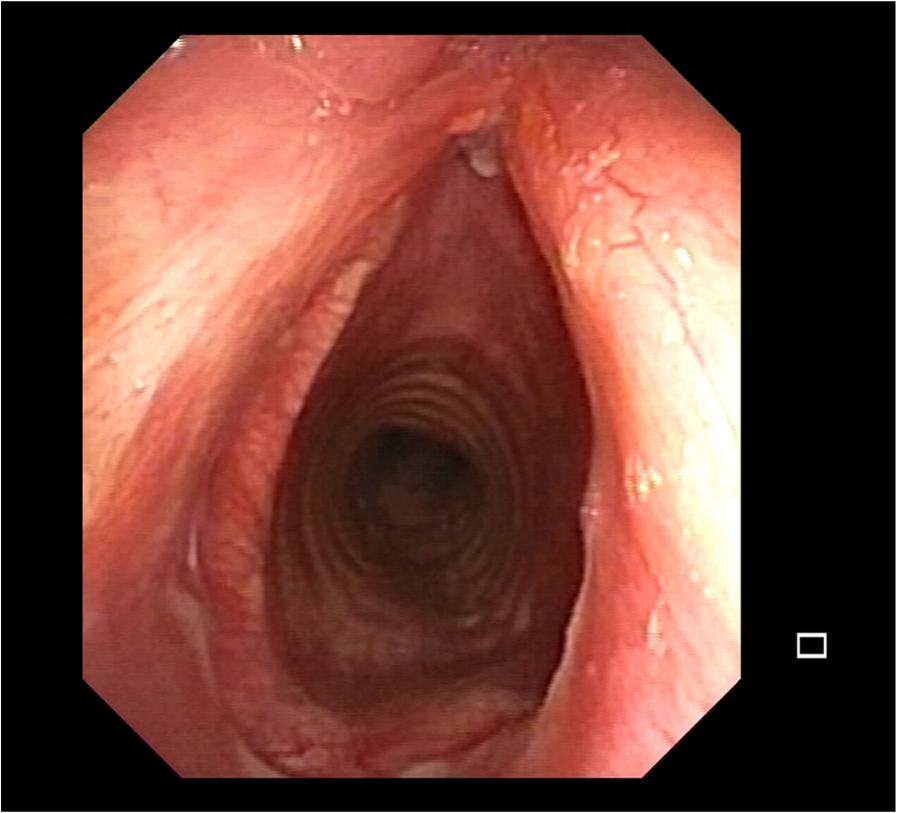
Follow up of the anastomosis which appear well consolidated

Technical failure following tracheal surgery is often related to anastomotic tension. Release maneuvers, including cervical tracheal mobilization, mediastinoscopic tracheal and bilateral bronchial release (MTBBR) [32], Dedo technique infrahyoid release [33, 34], Montgomery technique suprahyoid laryngeal release [35], hilar U-shaped release and division of the inferior pulmonary ligaments are often necessary in LTRR and TRR based on the extent of the tracheal stricture/neoplasm. After suturing approximately two-thirds of the circumference and before tying down the sutures, the head is mildly flexed to reduce anastomotic tension. It remains fixed with two strong chin-chest stitches in this position for 7–15 days postoperatively. At the end of the operation, the anastomosis is tested for air leaks. Most leaks require reperforming the anastomosis in order to avoid excessive trauma to the tracheal mucosa. Bronchoscopy is then performed to inspect the anastomosis visually and assess for technical problems such as loose sutures or bleeding (Fig. 4). This is repeated as necessary. Prior to hospital discharge, all patients should undergo a flexible bronchoscopy to examine the anastomosis. Any sign of early ischemia, necrosis, or leak must be recognized and treated. Follow up examination usually includes tracheo-bronchoscopy controls, with the timing depending on the patient and suture (Fig. 5).

## Complications and discussion of the literature

Tracheal resection is considered a relatively safe procedure if performed by an experienced surgeon. The overall success rate described in the literature for TRR and LTRR is > 95% [18, 36, 37]. Nevertheless the complication rate is still high (15–39%), even in the largest series [38, 39], and complications can be distinguished as non-anastomotic and anastomotic. Non anastomotic complications generally include wound infections and bleeding, glottic dysfunction and laryngeal edema but also can include pneumonia, myocardial infarction, arrhythmia, and pulmonary embolism. Anastomotic complications are granulation and restenosis (0–11%), dehiscence (0–5%), and fistula to surrounding structures such as the esophagus and innominate artery, even if extremly rare. Several authors have addressed anastomotic complications to the tension of the suture line, showing higher rate of early dehiscence or late restenosis [40]. When anastomotic complications occur, perioperative mortality and long term mordibity increase. The reoperation rate reported in the literature is 0–3%. (Table 1).

**Table 1:** The reoperation rate reported in the literature

Study	N	Resection Lenght, cm	Release, %	Major Complications, %	Anastomotic complications, %	Stenosis	Separation	Granulation	Mortality rate, %
Wright et al.,^8^2004	901	1–6.5, mean 3.3	9%	18.2%	9%	45,6%	45,6%	8,6%	1.2%
Piazza et al.,^38^ 2014	137	1.5–4, mean 2.7	_	38%	15%	38%	47,6%	14%	≤ 1%
Mutrie et al.,^21^ 2011	105	1.5–6, mean 2.7	None	34%	18%	17%	1%	_	1%
D’Andrilli et al.,^23^2015	109	1.5–6, mean 3.4 ± 0.8	8%	12,8%	9.2%%	80%	10%	–	0%
Bibas et al.,^22^2014	94	2.9 ± 0.83	–	44.6%	21%	16%	1%	4%	0%
Mohsen et al.,^39^2018	52	4.0–5.2, ≥ mean 43.78	85%	52%	13.4%	13.4%	0%	–	0%

The largest series reported in literature is the experience of Wright et al. [11] that analyzed their cohort of 901 patients treated with TRR or LTRR: 589 for postintubation tracheal stenosis, 208 for tumor, 83 for idiopathic laryngotracheal fistula and 21 for tracheoesophageal fistula. LTRR were performed in 281. The surgical approach was cervical (75%), mediastinal (20%) or thoracic (5%). Release were performed in 81 (9%). The median length of tracheal resection was 3.3 cm (range 1–6.5), ≥ 4 cm in 31%. Complications occured in 164 patients (18.2%) and were anastomotic complications in 81 (9%) (granulation, stenosis or separations). Predictors of anastomotic complications were reoperation, diabetes, lenght of resection ≥4 cm, laryngotracheal resection, young age and tracheostomy. The mortality among patients who had anastomotic complications was 7.4% whereas it was 0.01% in whom without. Furthermore, they asserted that, for patients undergoing reoperation, the failure rate increases for all resection lengths, except the shortest, and was double that for primary resections, probably due to peritracheal fibrosis generated after the previous operation and the higher risk of devascularization. Despite the high risk, reoperation can be successfully performed in well selected patients.

Piazza et al. [41] reviewed their cohort of 137 patients, all of whom had undergone resection for neoplastic or non-neoplastic disease except one (cervicotomy + sternotomy) through a cervicotomic approach and found a length of resection > 3.4 cm and preoperative tracheotomy to be a predictor of major surgical complications. No differences between benign or malignant resections were observed and no patients required laryngeal release.

Mutrie and colleagues [24] similarly described their experience on 105 patients. The median length of resected trachea was 2.7 cm. No patients required sternal division or laryngeal release. The complication rate was 17% and mortality 1% due to myocardial infarction.

D’Andrilli et al. [26] observed no postoperative mortality in their series of 109 patients who underwent laryngotracheal resection with primary end to end anastomoses. The anastomotic complications rate was 9.2% (10 patients) and included 8 restenosis, 1 dehiscence, and 1 glottic edema requiring tracheostomy. Non-anastomotic complications occurred in 4 patients (3 wound infections and 1 atrial fibrillation). The median length of the airway resection was 3.4 cm and in 14 patients the tracheal segment resected was longer than 4.5 cm. Release was performed on 9 patients (7 suprahyoid, 1 pericardial, and 1 suprahyoid plus pericardial release).

Bibas and associates [25] analyzed the results of 94 patients. The complication rate was 44.6% with no mortality and anastomotic complications in 21% of patients: 16% restenosis, 4% granulation tissue, and 1% dehiscence. The most frequent non-anastomotic complication observed was superficial wound infection (10.6%). The median resection length was 2.9 cm and the complication rate was higher in tracheal resectionss> 3.63 cm in length. However, the most prominent risk factor in that series seen in multivariate analysis was a previous history of tracheal resection.

Mohsen et al. [42] recently described their experience of 52 long-tracheal segments resection (40-54 mm). The most common cause of resection was postintubation stricture followed by neoplastic lesions. All patients underwent TRR through a collar incision, in 13 cases a manubrotomy was necessary too. They performed 26 cricotracheal anastomosis, 22 tracheotracheal anastomosis and 4 thyrocricotracheal anastomosis. Release manoeuvres were used in 85% of patients to achieve tension free anastomosis, the most used in long segment resection was the MTBBR (19/52). The following complications were observed: swallowing and phonation dysfunction were detected in 17 (32.2%) while restenosis in 7 (13.4%) patients. The overall success rate was 86.3% at 5 years with no mortality, but there were no patients with previous resection.

## Conclusions

Despite whether the nature of primary disease is benign or neoplastic, laryngotracheal and tracheal resection and reconstruction have proved to be safe procedures and the success rate is high. Even when complications occur, the morbidity rate is 45%. The best treatment for complications is prevention and it is clear from the literature that previous tracheal surgery and resections > 4 cm long are associated with a consistent increase of the failure rate. Adequate mobilization of the proximal and distal trachea is necessary to achieve a tension free anastomosis, which is the goal of tracheal surgery. Thus, use of a release procedure or definitive stent treatment should be considered in patients undergoing resections > 4 cm. Accurate selection of patients focused on the presence of comorbidities also is fundamental.

## Abbreviations

HFJV: High frequency jet ventilation
LTRR: Laryngotracheal
MTBBR: Mediastinoscopic tracheal and bilateral bronchial release
TRR: Tracheal resection and reconstruction
